# Roles of cytokine storm in sepsis progression: biomarkers, and emerging therapeutic strategies

**DOI:** 10.3389/fimmu.2025.1696366

**Published:** 2025-11-04

**Authors:** Weibin You

**Affiliations:** Department of Critical Care Medicine, West China Hospital of Sichuan University, Chengdu, China

**Keywords:** cytokine, sepsis, biomarker, immune response, therapy, inflammatory cell death

## Abstract

Sepsis is a life-threatening syndrome marked by uncontrolled systemic inflammation, cytokine storm, and organ dysfunction. Central to its pathogenesis is innate immune hyperactivation, which triggers excessive cytokine release and inflammatory cell death, ultimately driving multiorgan failure. Despite advancements in intensive care, immune dysregulation remains a major therapeutic hurdle. Moreover, recent discoveries of emerging biomarkers, such as serum amyloid A (SAA), high-density lipoprotein (HDL), monocyte distribution width (MDW), neutrophil-to-lymphocyte ratio (NLR), and RDW-to-albumin ratio (RAR), highlight their potential diagnostic and prognostic value. This review systematically summarizes the cellular and molecular mechanisms underlying cytokine storm, emphasizing the roles of TNF-α, IL-1β, IL-6, and inflammasome activation. Furthermore, we outline current and emerging therapeutic strategies targeting both immune overactivation and late-stage immunosuppression, including cytokine antagonists, immune checkpoint inhibitors, and nanomedicine-based approaches, providing a comprehensive framework to guide precision immunotherapy in sepsis management.

## 1 Introduction

Sepsis is a life-threatening clinical syndrome stemming from a dysregulated immune response to infection, marked by systemic inflammation and organ dysfunction (1). Its pathogenesis involves the recognition of pathogen-associated molecular patterns (PAMPs) and damage-associated molecular patterns (DAMPs) via pattern recognition receptors (PRRs), initiating innate immune activation and excessive cytokine production (2). Although antimicrobial therapy and organ support are essential, they have shown limited efficacy in reducing mortality, underscoring the need to target immune dysregulation. Central to this process is the cytokine storm, driven by uncontrolled release of TNF-α, IL-1β, and IL-6, which exacerbates systemic inflammatory response syndrome (SIRS) and tissue injury (3).

Amplification of inflammation is further fueled by pyroptosis and necroptosis, releasing intracellular DAMPs that perpetuate the immune cascade and lead to multiple organ dysfunction syndrome (MODS) (4). Septic shock, the most severe form, carries a mortality rate of up to 70% (5), necessitating early and precise intervention. Hence, identifying biomarkers for early diagnosis, risk stratification, and prognostic assessment is crucial (6). This review delineates the mechanistic basis of cytokine storm-driven injury in sepsis, explores evolving immunomodulatory therapies, and evaluates emerging diagnostic tools to inform clinical decision-making.

## 2 Mechanisms underlying the cytokine storm in sepsis

### 2.1 Cytokine-driven hyperinflammation in sepsis

Sepsis results from a profoundly dysregulated host immune response to infection, in which homeostatic immune control is lost. Unlike localized infections, sepsis manifests as an unrestrained systemic inflammatory cascade, dominated by the overproduction of pro-inflammatory cytokines. Central mediators of this cascade include tumor necrosis factor-α (TNF-α), interleukins (IL-1, IL-6, IL-12), interferons (IFN-α, IFN-β, IFN-γ), monocyte chemoattractant protein-1 (MCP-1), and IL-8, each contributing to the propagation of inflammation and the onset of a cytokine storm—characterized by immune hyperactivation, extensive tissue injury, and multi-organ dysfunction (7). This hyperinflammatory state is driven primarily by two converging mechanisms: hypersensitivity of innate immune sensors and the induction of inflammatory cell death pathways. Pattern recognition receptors (PRRs) detect pathogen-associated molecular patterns (PAMPs) and damage-associated molecular patterns (DAMPs), triggering downstream signaling cascades such as NF-κB and AP-1, which upregulate pro-inflammatory gene programs (8). Central to this process are inflammasomes—multiprotein complexes that sense intracellular stress and activate caspase-1, which in turn cleaves pro-IL-1β and pro-IL-18 into their active forms. Although acute cytokine responses can support pathogen clearance and tissue repair, their dysregulation results in sustained hypercytokinemia, which disrupts immune equilibrium, induces host tissue toxicity, and initiates a self-amplifying cycle of inflammation and immunopathology (9).

### 2.2 Inflammatory cell death

Inflammatory forms of programmed cell death (PCD) are central to the pathophysiology of cytokine storm in sepsis. Among the PCD pathways, pyroptosis and necroptosis—unlike the immunologically silent apoptosis—are inherently pro-inflammatory and thus critically implicated in disease progression. Pyroptosis is executed through gasdermin family pore formation, while necroptosis is driven by RIPK3-mediated oligomerization of MLKL, resulting in membrane rupture and subsequent DAMP release (10). Although these modalities were once viewed as discrete, mounting evidence reveals substantial crosstalk, particularly under sustained inflammatory stress. This interplay culminates in panoptosis, a unified death pathway integrating molecular components of pyroptosis, apoptosis, and necroptosis (11). Notably, innate immune responses to pathogens such as SARS-CoV-2 elicit high levels of TNF-α and IFN-γ, which act synergistically to amplify panoptosis. Murine models provide compelling evidence of its pathogenic role, as pharmacologic inhibition of panoptosis significantly lowers mortality in cytokine-driven sepsis, establishing a mechanistic link between excessive cytokine production and inflammatory cell death. Moreover, PAMPs, DAMPs, and pro-inflammatory cytokines form a self-amplifying circuit, whereby inflammation promotes further immune cell death, and the products of dying cells exacerbate cytokine release, sustaining a vicious cycle of immune dysfunction (12).

### 2.3 Evolution of cytokine storm in advanced sepsis

Although advances in intensive care have improved early survival in CS and septic shock, persistent immune and tissue dysfunction remain barriers to recovery. While acute-phase sepsis has received significant focus, long-term outcomes are often dictated by sustained immunological imbalance. In late-stage sepsis, CS disrupts Th1/Th2 balance, impairing antimicrobial defense and promoting autoimmune tissue damage (13). Concurrently, excessive reactive oxygen species (ROS) and circulating cell-free DNA (cfDNA) amplify inflammation via PRRs, especially Toll-like receptors (TLRs), perpetuating disease progression (14). Early lymphocyte depletion, a hallmark of sepsis, correlates with increased mortality (15). Sepsis also skews hematopoiesis toward monocyte and neutrophil expansion, promoting immature myeloid-derived suppressor cell (MDSC) accumulation and anti-inflammatory cytokine secretion. Simultaneously, reduced expression of HLA-DR on antigen-presenting cells impairs pathogen recognition and Th1/Th2 signaling, worsening immune dysfunction (16). A subset of patients progresses to persistent inflammation–immunosuppression–catabolism syndrome (PICS), marked by chronic inflammation, immune suppression, hematopoietic dysregulation, muscle wasting, and poor functional recovery, often requiring prolonged ICU care (17). Clinically, PICS manifests as ventilator dependence, secondary infections, ICU-acquired weakness, and rehospitalization (18). Many fail to regain baseline function, requiring long-term care. The chronic immune dysfunction in PICS complicates therapy, limiting the efficacy of standard antimicrobials or immunosuppressants. Importantly, early identification using markers such as lymphopenia, low HLA-DR, and elevated IL-6, coupled with immunostimulatory therapy, nutrition, and rehabilitation, is vital for improving outcomes (19).

## 3 Biomarkers in sepsis

### 3.1 Serum amyloid A

Serum amyloid A (SAA) is a prototypical acute-phase reactant predominantly synthesized by hepatocytes in response to systemic inflammatory stimuli. Circulating in complex with high-density lipoproteins (HDL), SAA modulates innate immunity via engagement with toll-like receptors (TLRs) and formyl peptide receptor-like molecules, thereby initiating proinflammatory cascades. Notably, SAA functions as a potent chemoattractant, directing neutrophils and monocytes to sites of inflammation, and serves as a sentinel biomarker across infectious and autoimmune disorders. In sepsis, elevated plasma SAA concentrations reflect the intensity of systemic inflammation and are markedly amplified during septic shock, correlating with disease severity and aiding prognostication (20, 21). Baseline serum levels range from 1–5& μg/mL under physiological conditions (22), but levels can surge up to a thousand-fold within hours of microbial insult, before declining upon pathogen clearance. This rapid kinetic profile underscores its sensitivity to inflammatory stress; however, its limited specificity diminishes its utility as a stand-alone diagnostic tool (23). Nonetheless, SAA remains clinically valuable, particularly in ruling out bacterial infection and guiding antimicrobial therapy. When combined with other acute-phase markers such as C-reactive protein (CRP), diagnostic accuracy improves for distinguishing bacterial etiologies (24). In a multicenter cohort, Li et al. (25) demonstrated that SAA, CRP, and procalcitonin (PCT) independently predicted sepsis risk, and that their integration into composite diagnostic panels significantly enhanced precision in critically ill populations.

### 3.2 High-density lipoprotein

High-density lipoprotein (HDL), composed of cholesterol, phospholipids, and apolipoproteins, orchestrates reverse cholesterol transport by shuttling peripheral cholesterol to the liver for excretion& (26). During sepsis, this homeostatic mechanism is severely compromised. Inflammatory insults precipitate both quantitative reductions in HDL levels and qualitative structural alterations, including particle enlargement and functional derangement& & (27). These modifications impair HDL’s vasoprotective roles, including its anti-inflammatory, antioxidant, and anticoagulant properties. Moreover, key HDL-associated enzymes—phospholipid transfer protein (PLTP) and cholesteryl ester transfer protein (CETP)—undergo dysregulation under septic conditions, further compromising lipid transport and immune modulation& (28). Therapeutic interventions aiming to enhance apolipoprotein A-I expression or inhibit PLTP/CETP activity have shown potential in restoring HDL functionality. Notably, oxidative modification of HDL disrupts its ability to regulate coagulation, correlates with heightened mortality risk, and demonstrates superior prognostic accuracy over conventional scoring systems such as APACHE II and SOFA in septic shock& & (29).

### 3.3 Monocyte distribution width

Monocyte Distribution Width (MDW) has emerged as a promising and cost-efficient biomarker for the early detection of sepsis, reflecting monocyte size heterogeneity through measurements of cell volume, conductivity, and light scatter properties. In early septic states, pathogen-associated molecular patterns (PAMPs) drive monocyte functional reprogramming, increasing morphological and phenotypic diversity within the circulating pool (30). Multiple studies have demonstrated that MDW possesses favorable diagnostic sensitivity and specificity (31), showing performance on par with conventional biomarkers in acute care settings (32). Agnello et al. (33) reported robust discriminatory power of MDW in emergency departments, where elevated values were consistently associated with microbiologically confirmed sepsis. A diagnostic threshold of 23.5 was identified as optimal, balancing sensitivity and specificity. However, the clinical adoption of fixed cutoffs such as MDW& >& 23.5 is hindered by variability introduced by patient demographics, comorbidities, and differences in instrumentation across healthcare systems, thereby complicating universal standardization. Despite these limitations, MDW has demonstrated superior diagnostic performance compared to procalcitonin (PCT) in some studies, suggesting its potential utility as a frontline screening tool for sepsis in high-throughput clinical environments (34, 35). Meta-analyses further corroborate its diagnostic value, indicating metrics comparable to CRP and PCT. When incorporated into multi-marker panels, MDW may significantly enhance diagnostic accuracy and risk stratification in sepsis (36).

### 3.4 Neutrophil-to-lymphocyte ratio

In sepsis, immune dysregulation triggered by microbial invasion can escalate into a cytokine storm, underscoring the need for reliable immunological markers to guide individualized interventions (37). Among peripheral indicators, the neutrophil-to-lymphocyte ratio (NLR) has emerged as a clinically accessible and dynamic metric, capturing the balance between innate immune activation and adaptive suppression (38). Its prognostic relevance in sepsis is increasingly recognized (39), with suggested thresholds ranging from 3 to 5 (40). Specifically, in elderly diabetic patients with sepsis, an NLR ≥3.482 was associated with adverse outcomes (41). Biologically, neutrophilia in sepsis reflects IL-6–driven emergency granulopoiesis, mobilizing neutrophils that enhance microbial clearance but concurrently exacerbate systemic inflammation via degranulation and ROS production (42, 43). Conversely, lymphopenia results from T cell apoptosis and exhaustion, facilitated by persistent cytokine exposure (TNF-α, IL-10) and upregulation of immune checkpoints such as PD-1 and CTLA-4, impairing adaptive responses (44–46). Compared to conventional markers including procalcitonin (PCT), ALT/AST ratio, and platelet-to-lymphocyte ratio (PLR), NLR may offer superior prognostic performance in specific clinical contexts (47). For instance, Zhong et al. (48) demonstrated that baseline NLR predicted disease progression in pediatric sepsis, and its combination with PCT improved early diagnostic sensitivity. Li et al. (49) identified both NLR and the monocyte-to-HDL ratio (MHR) as independent predictors of 28-day mortality. Similarly, Wei et al. (50) associated elevated NLR with increased mortality in septic patients complicated by acute myocardial infarction. While NLR offers operational simplicity, its prognostic utility improves when integrated with other biomarkers and monitored dynamically—an approach that aligns with the complex and evolving nature of sepsis pathobiology.

### 3.5 Prognostic utility of the red cell distribution width-to-albumin ratio in sepsis

**Figure 1:**
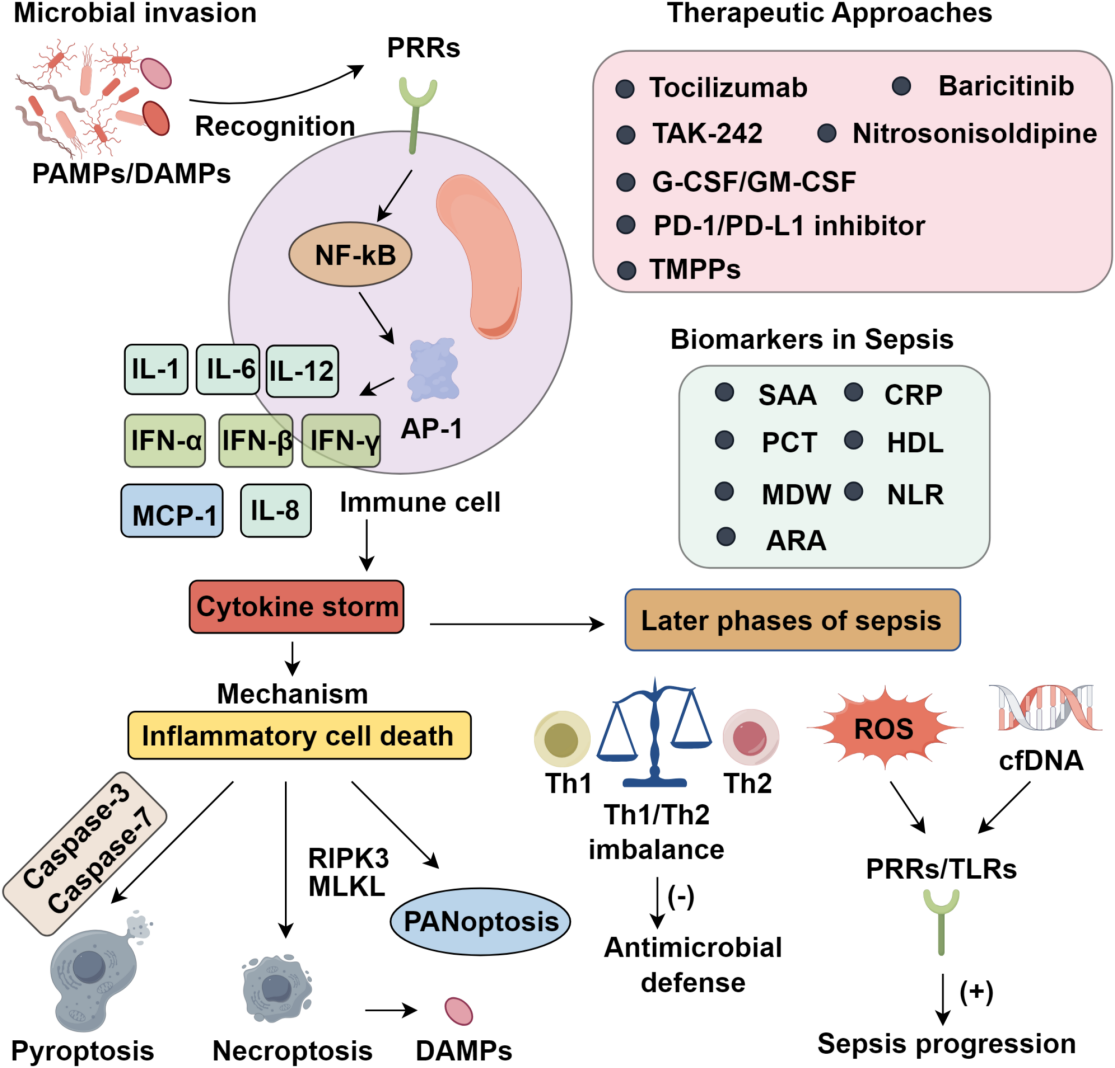
Roles of cytokine storm in sepsis progression.

Red cell distribution width (RDW), an indicator of anisocytosis, reflects variability in erythrocyte size and is elevated across a range of pathological states. In sepsis, increased RDW has been robustly associated with adverse outcomes, and proposed cutoff values facilitate early risk stratification. When combined with serum lactate, RDW achieves diagnostic performance comparable to SOFA and APACHE II scores (51). Although RDW lacks high specificity, Moisa et al. (52) demonstrated its relevance in bacterial sepsis, where elevations reflect inflammation-induced disruption of erythropoiesis and red blood cell turnover. Albumin (ALB), a negative acute-phase reactant synthesized in the liver, maintains oncotic pressure and exerts antioxidant and anti-apoptotic effects at the endothelial interface. During sepsis, albumin’s detoxification and toxin-binding functions are markedly impaired (53). Alterations in baseline ALB, its dynamic fluctuations, and minimal values independently correlate with mortality risk (54, 55). When combined with CRP and pre-existing functional status, ALB reliably predicts 28-day mortality in elderly septic patients, with accuracy comparable to SOFA scores (56). Recently, the RDW-to-albumin ratio (RAR) has emerged as a composite biomarker with prognostic value across inflammatory disorders, including pneumonia, hepatic cirrhosis, and heart failure. In sepsis, Xu et al. (57) demonstrated that RDW and ALB reflect distinct pathophysiological axes—hematopoietic disruption and systemic inflammation, respectively. By integrating these divergent processes, RAR provides improved predictive power for short-term morbidity and mortality, underscoring its potential clinical utility (**Figure 1**).

## 4 Therapeutic approaches for controlling systemic inflammation in sepsis

### 4.1 Pharmacologic regulation of inflammatory dysregulation

In the early stages of sepsis, a systemic cytokine storm (CS) driven by excessive proinflammatory mediator release represents a key therapeutic target. Pharmacological strategies thus focus on attenuating this initial hyperinflammatory cascade to limit tissue injury and organ failure. In preclinical models, blockade of major cytokines such as TNF-α and IFN-γ significantly improves survival outcomes (58). However, clinical trials targeting TNF-α, IL-1β, and other mediators have yielded limited success—largely due to immune heterogeneity among patients, suboptimal timing of intervention, and the pleiotropic roles of these cytokines in host defense. Tocilizumab, an IL-6R monoclonal antibody, has demonstrated efficacy in modulating cytokine dysregulation during COVID-19–related sepsis, highlighting its broader potential in inflammatory syndromes (58). Upstream inhibition using Janus kinase (JAK) inhibitors, such as baricitinib, offers more comprehensive suppression by concurrently modulating IL-6, IL-1β, and TNF-α signaling and reducing compensatory pathway activation (59). Targeting pathogen recognition pathways, particularly Toll-like receptor 4 (TLR4), has also emerged as a viable approach. TLR4 mediates lipopolysaccharide (LPS)-induced inflammatory signaling; its inhibition reduces cytokine production and mitigates tissue damage in sepsis models (60). The small-molecule inhibitor Resatorvid (TAK-242) attenuates organ dysfunction and improves survival in animal studies (61), though its lack of antimicrobial activity underscores the need for combinatory or sequential approaches. Caspases, particularly caspase-1, regulate both inflammasome activation and pyroptosis. Inhibition of caspase-1 suppresses NLRP1 inflammasome signaling, gasdermin D (GSDMD) cleavage, and the maturation of IL-1β and IL-18, thereby ameliorating sepsis-induced acute kidney injury (62). While no caspase inhibitor has gained clinical approval, nitrosonisoldipine—a photolytic metabolite of nisoldipine—selectively inhibits caspases-1, -4, and -11. It effectively blocks pyroptosis and dampens IL-1β release, improving survival in murine models. These findings support further development of caspase inhibitors as therapeutic candidates in sepsis.

### 4.2 Pharmacological modulation of immunosuppression

#### 4.2.1 Cytokine-based immunostimulatory therapies

Although advances in understanding sepsis pathophysiology and critical care have reduced early multiple organ dysfunction, late-stage mortality remains predominantly driven by profound immune suppression. This has shifted therapeutic strategies toward preventing immune cell apoptosis and reversing immunosuppression. Interleukin-7 (IL-7), noted for its robust anti-apoptotic activity, promotes T cell survival and proliferation, thereby restoring adaptive immunity. Both preclinical and clinical studies support the use of recombinant IL-7 in septic patients, demonstrating recovery of lymphocyte counts and improved immune competence without triggering systemic hyperinflammation or organ toxicity (63). Moreover, granulocyte colony-stimulating factor (G-CSF) and granulocyte-macrophage colony-stimulating factor (GM-CSF) have exhibited the capacity to counteract sepsis-induced immune paralysis. In particular, GM-CSF has been shown to enhance human leukocyte antigen-DR (HLA-DR) expression on circulating monocytes, thereby augmenting microbial clearance efficiency (19). In parallel, targeting Toll-like receptors such as TLR2 and TLR4 to modulate the secretion of pro-inflammatory cytokines presents another viable approach to facilitate immune restoration (64).

#### 4.2.2 Therapeutic blockade of immune checkpoint molecules

Immune checkpoint regulators serve as key negative modulators of T lymphocyte activation and cytokine secretion during the adaptive immune phase& (65). Among them, the PD-1/PD-L1 axis plays a pivotal role in modulating the magnitude and duration of immune responses. Pharmacological blockade of PD-1 or PD-L1 has been shown to reinvigorate exhausted T cells and restore antimicrobial immunity in preclinical and clinical sepsis models, with evidence of enhanced T cell functionality and improved survival (66). However, the use of immune checkpoint inhibitors (ICIs) in sepsis necessitates caution, given their potential to trigger immune-related adverse events. Thus, patient stratification based on immunological status is paramount. Low HLA-DR expression on monocytes—indicative of immunoparalysis and associated with adverse prognosis—may help identify candidates likely to benefit from ICI therapy (67). Likewise, elevated PD-1 levels on CD4^+^ and CD8^+^ T cells suggest functional exhaustion and can inform therapeutic eligibility (45). Recent advancements in immune profiling, including multiparameter flow cytometry and transcriptomic platforms, enable dynamic assessment of immune competence. These tools provide actionable insights for balancing efficacy with safety during ICI administration (68). Overall, these findings underscore the promise of immune checkpoint inhibition as a targeted strategy to reverse sepsis-induced immunosuppression and improve clinical outcomes.

#### 4.2.3 Therapeutic applications of nanotechnology

Emerging nanotechnological platforms have shown considerable promise in counteracting the immunopathology of sepsis, particularly in attenuating cytokine storm–associated inflammation (69). One notable innovation is the development of tannic acid–Mn²^+^–polymyxin B–PVP nanoparticles (TMPPs), which integrate antimicrobial and anti-inflammatory functionalities. Polymyxin B (PMB) enhances bactericidal activity while neutralizing lipopolysaccharide (LPS), thereby disrupting TLR4-mediated signaling and suppressing pro-inflammatory cytokine production. Concurrently, tannic acid (TA) scavenges reactive oxygen species (ROS) and neutralizes cell-free DNA (cfDNA), both key drivers of CS-induced tissue injury (70). This multifunctional design addresses the shortcomings of single-target agents like TAK-242, which lack intrinsic antimicrobial effects. TMPPs exhibit superior efficacy in mitigating pulmonary and systemic inflammation in septic models, underscoring the therapeutic potential of nanomedicine to modulate hyperinflammatory states and restore immune balance.

## 5 Conclusion

Sepsis represents a paradigm of immune dysregulation, where hyperinflammatory responses evolve into profound immunosuppression, driven in part by sustained CS and inflammatory cell death. Unchecked cytokine release not only promotes multiorgan failure but also reshapes the immune landscape through pyroptosis, necroptosis, and PANoptosis, perpetuating a vicious cycle of damage. Understanding these intertwined mechanisms has revealed critical molecular targets, offering new therapeutic avenues that may disrupt this pathological feedback loop. In parallel, the identification of reliable biomarkers such as SAA, HDL, MDW, NLR, and RAR enhances early diagnosis, risk stratification, and therapeutic monitoring. Additionally, emerging interventions—including cytokine blockade, immune checkpoint modulation, and nanotechnology-driven delivery systems—highlight the promise of precision immunomodulation in sepsis care. Future strategies should aim to integrate multi-biomarker panels with individualized therapies to balance inflammation and immune competence. Collectively, translating these mechanistic insights into clinical practice may transform the current sepsis paradigm, shifting from reactive care to targeted immunological control.
